# Anesthetic Sevoflurane Causes Rho-Dependent Filopodial Shortening in Mouse Neurons

**DOI:** 10.1371/journal.pone.0159637

**Published:** 2016-07-21

**Authors:** Jeffrey H. Zimering, Yuanlin Dong, Fang Fang, Lining Huang, Yiying Zhang, Zhongcong Xie

**Affiliations:** 1 University of Rochester School of Medicine and Dentistry, Rochester, New York, United States of America; 2 Geriatric Anesthesia Research Unit, Department of Anesthesia, Critical Care and Pain Medicine, Massachusetts General Hospital and Harvard Medical School, Charlestown, Massachusetts, United States of America; 3 Howard Hughes Medical Institute, Harvard Medical School, Boston, Massachusetts, United States of America; Imperial College London, Chelsea & Westminster Hospital, UNITED KINGDOM

## Abstract

Early postnatal anesthesia causes long-lasting learning and memory impairment in rodents, however, evidence for a specific neurotoxic effect on early synaptogenesis has not been demonstrated. Drebrin A is an actin binding protein whose localization in dendritic protrusions serves an important role in dendritic spine morphogenesis, and is a marker for early synaptogenesis. We therefore set out to investigate whether clinically-relevant concentrations of anesthetic sevoflurane, widely- used in infants and children, alters dendritic morphology in cultured fetal day 16 mouse hippocampal neurons. After 7 days in vitro, mouse hippocampal neurons were exposed to four hours of 3% sevoflurane in 95% air/5% CO_2_ or control condition (95% air/5% CO_2_). Neurons were fixed in 4% paraformaldehyde and stained with Alexa Fluor555-Phalloidin, and/or rabbit anti-mouse drebrin A/E antibodies which permitted subcellular localization of filamentous (F)-actin and/or drebrin immunoreactivity, respectively. Sevoflurane caused acute significant length-shortening in filopodia and thin dendritic spines in days-in-vitro 7 neurons, an effect which was completely rescued by co-incubating neurons with ten micromolar concentrations of the selective Rho kinase inhibitor Y27632. Filopodia and thin spine recovered in length two days after sevoflurane exposure. Yet cluster-type filopodia (a precursor to synaptic filopodia) were persistently significantly decreased in number on day-in-vitro 9, in part owing to preferential localization of drebrin immunoreactivity to dendritic shafts versus filopodial stalks. These data suggest that sevoflurane induces F-actin depolymerization leading to acute, reversible length-shortening in dendritic protrusions through a mechanism involving (in part) activation of RhoA/Rho kinase signaling and impairs localization of drebrin A to filopodia required for early excitatory synapse formation.

## Introduction

Early postnatal anesthesia causes long-lasting learning and memory impairment in rodents [1], and observational and prospective human studies suggest an increased risk for learning and memory impairment in children exposed to anesthesia before the age of 3–4 years old [2], although the short exposure to anesthesia may not lead to cognitive impairment [3]. Since millions of children undergo general anesthesia worldwide each year, elucidation of the mechanisms underlying anesthetic-induced neurotoxicity in developing brain is not only of general scientific interest, but may also have substantial public health significance.

Sevoflurane, a commonly used anesthetic in children, has been shown to induce apoptosis [4,5], neuroinflammation [6], Tau phosphorylation [7], as well as cognitive impairment [5–8] in young mice. Synapses mediate long-term memory in the brain; dendritic spines are the anatomical locus of most excitatory synapses in the central nervous system [9]. In a prior study, sevoflurane administered to postnatal day 7 rodents caused learning impairment associated with decreased synaptogenesis [10]. Thus we set out (in the present study) to assess the morphologic effects of sevoflurane on dendritic spine or filopodia formation in early developing mouse hippocampal neurons in culture. The goal of our studies is to establish a model in neurons to elucidate the underlying mechanism by which sevoflurane affects synaptogenesis during a critical period in brain development.

Dendritic spines are dynamic structures comprised of clusters of postsynaptic neurotransmitter receptors and cytoskeletal components, e.g. actin monomers and actin filaments, i.e. F-actin [11]. Dendritic spine formation and disappearance is modulated by experience-dependent and experience-independent electrical activity in a network of neurons [11]. Although the precise mechanisms are not completely understood, it is generally accepted that cytoskeletal changes involving actin (occurring on a timescale ranging from seconds to days or weeks) are linked to ongoing neuronal activity which regulates dendritic spine formation (favoring synaptogenesis) or spine elimination (favoring synapse elimination) [12].

Isoflurane, a closely-related volatile anesthetic, has been shown to impair actin dynamics by altering activity in RhoA- GTPases leading to decreased neuritic projections and apoptosis in cultured mouse hippocampal neurons [13]. Filopodia and thin spines are highly motile, immature dendritic protrusions which serve as precursors to mature, larger-head spines. The latter contain the synaptic machinery necessary for stable, long-term memory [11]. We tested the possibility that sevoflurane can alter the appearance and disappearance of filopodia and thin spines in early developing hippocampal neurons via effects on RhoA/Rho associated kinase (ROCK)-mediated F-actin depolymerization. Our hypothesis is that anesthetic sevoflurane decreases the length of filopodial and thin spine in 7 days in vitro (DIV) neurons.

## Materials and Methods

### Mice

This study was carried out in strict accordance with the recommendations in the Guide for the Care and Use of Laboratory Animals of the National Institutes of Health. The animal protocol was approved by the Massachusetts General Hospital (Boston, Massachusetts) Standing Committee on the Use of Animals in Research and Teaching (Protocol Number: 2006N000219).

Animals were kept in a temperature-controlled (22–23°C) room under a 12-h light/dark period (light on at 7:00 AM); standard mouse chow and water were available ad libitum. Housing was provided with appropriate tactile, olfactory, visual, and auditory stimuli. No more than two adults were housed together in a cage when litter was born. Pups were weaned by 21–28 days of age. Every effort was made to minimize the number of mice that were used in experiments. Two C57BL/6J female mice (The Jackson Laboratory, Bar Harbor, ME) were mated with one male mouse. The pregnant mice were identified and then housed individually. At gestation stage day 16 (G16), the pregnant mice were euthanized by carbon dioxide with an increasing concentration of 20% of the chamber volume/minute. All efforts were made to minimize suffering of the animals.

### Primary Neurons

Fetuses were removed by caesarean section and transferred to a sterile petri dish containing phosphate-buffered saline (PBS) [14]. Hippocampi were dissected from surrounding tissues, and the meninges were removed. The neurons were dissociated by trypsinization and trituration as previously described [14]. The dissociated cells were re-suspended in serum-free B27/neurobasal medium and plated at 100,000 cells/mL on 35mm Fluoro-dishes (World Precision Instruments, Sarasota, FL) that had been pre-coated with poly-D-lysine, (Sigma, St. Louis, MO) and laminin (Thermo- Fisher Scientific, Waltham, MA). Seven days after plating, the cultures, which contained virtually pure neurons, were exposed to sevoflurane.

### Anesthetic Treatment

The anesthesia treatment was performed as described in our previous studies [15]. Inhalation anesthetic sevoflurane was delivered from an anesthesia machine to a sealed plastic box in a 37 degrees C incubator containing 35 mm dishes or six-well plates seeded with 100,000 neurons/mL of serum-free B27/neurobasal medium. A Datex infrared gas analyzer (Puritan-Bennett, Tewksbury, MA) was used to continuously monitor the delivered concentrations of carbon dioxide, oxygen, and sevoflurane. The cells were treated for four hours with sevoflurane alone (21% O_2_, 5% CO_2_ and 3% sevoflurane). The control condition for sevoflurane was 21% O_2_ plus 5% CO_2_.

### Immunocytochemistry

Control or sevoflurane-treated DIV 7 primary neurons were either fixed in 4% paraformaldehyde immediately following anesthesia or maintained for 2 additional days in culture prior to staining with Alexa Fluor 555 Phalloidin from Invitrogen (5 units/mL), specific for filamentous F-actin. Neuron fixation in 4% paraformaldehyde in PBS was performed at room temperature for 20 minutes. The fixed neurons were next permeabilized with freshly prepared 0.1% Triton X-100 in PBS at room temperature for 5 minutes, followed by a 5 minute PBS wash; the permeabilization/wash was repeated three times. Next, the cells were blocked with 1% bovine serum albumin (BSA) in PBS at room temperature for 30 minutes. Finally, the cultures were incubated with Alexa Fluor 555 Phalloidin for 20 minutes at room temperature, washed with PBS, and a drop of Vectashield (Vector Laboratories, Burlingame, CA) was added prior to mounting on glass slides for imaging.

### Chemicals

Y27632 and MTT were obtained from Sigma (St. Louis, MO). Alexa Fluor 555 Phalloidin (A34055) was obtained from Invitrogen (Grand Island, NY).

### Antibodies

Rabbit anti-mouse drebrin A/E polyclonal primary antibody (AB10140, 1:100) was obtained from EMD Millipore (Billerica, MA). Alexa Fluor 488 goat anti-rabbit polyclonal secondary antibody (A11008, 1:500) was obtained from Invitrogen (Grand Island, NY).

### Fluorescence Imaging

**Fig 1:**
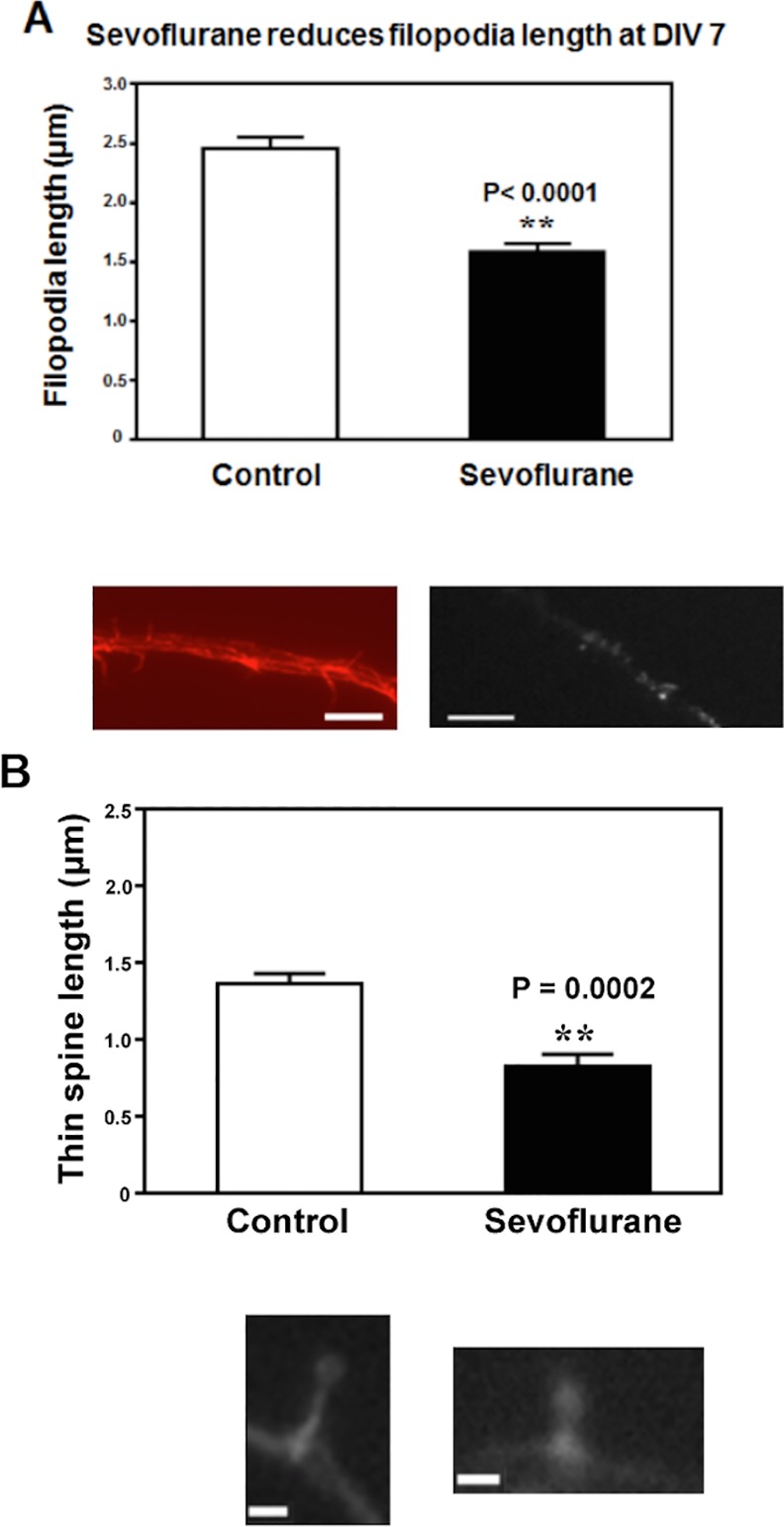
**Sevoflurane reduces A) filopodia and B) thin spine length in DIV7 immature mouse hippocampal neurons.** (A) 3% sevoflurane for four hours reduced mean filopodia length in hippocampal neurons immediately after the exposure. (B) 3% sevoflurane for four hours reduced mean thin spine length in hippocampal neurons immediately after the exposure. (**: the difference between sevoflurane-exposed and unexposed neurons.) Representative fluorescent images of (phalloidin-stained) F-actin containing, control or sevoflurane-exposed dendritic branches show acute length-shortening of A) filopodia or B) thin spines following sevoflurane treatment. N = 167 protrusions, similar results were obtained in three experiments. Calibration bars are 5 microns (A) and 0.5 microns (B) in length.

**Fig 2:**
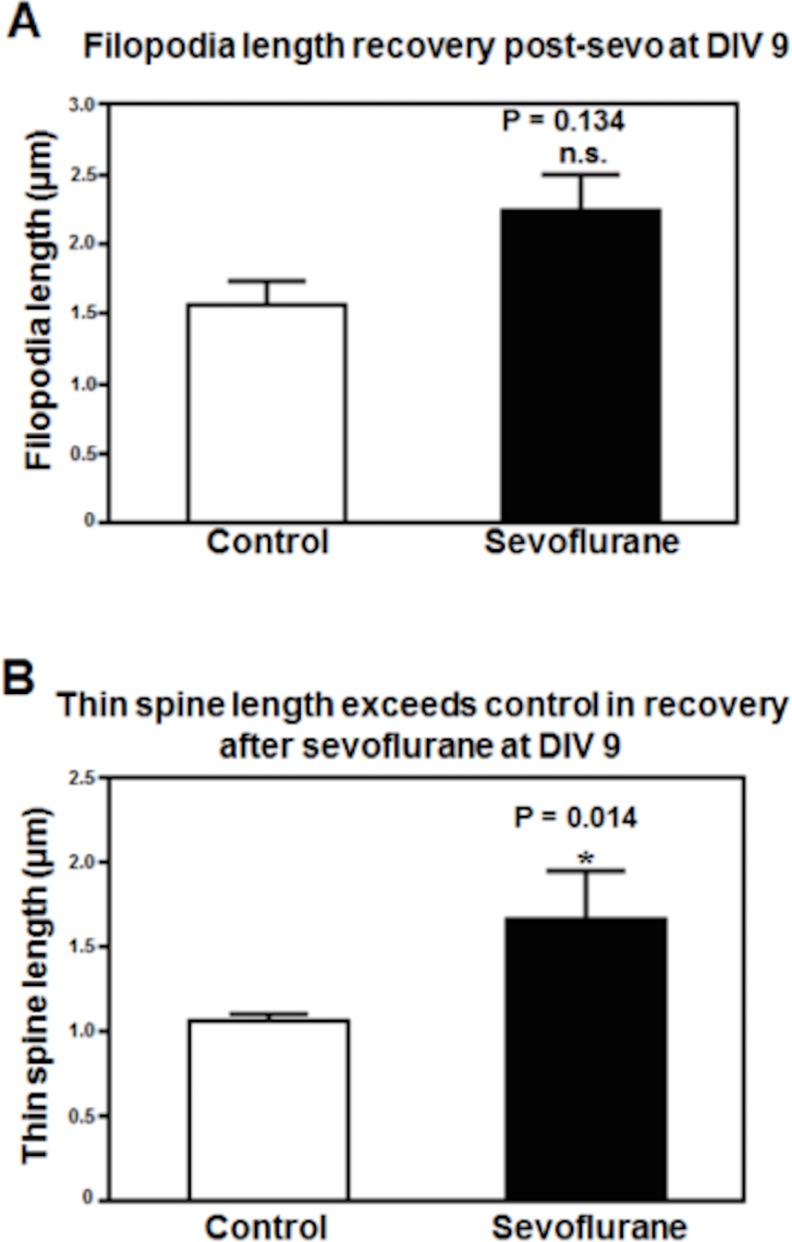
Recovery of filopodia length 2 days after sevoflurane exposure on DIV7. (A) Two days after acute sevoflurane exposure, filopodia length recovered to mean levels observed in control unexposed DIV9 hippocampal neurons. (B) Two days after acute sevoflurane exposure, thin spine length exceeded the mean level in control unexposed DIV9 hippocampal neurons. (*: the difference between sevoflurane-exposed and unexposed neurons.) N = 76 protrusions, similar results were obtained in three experiments.

**Fig 3:**
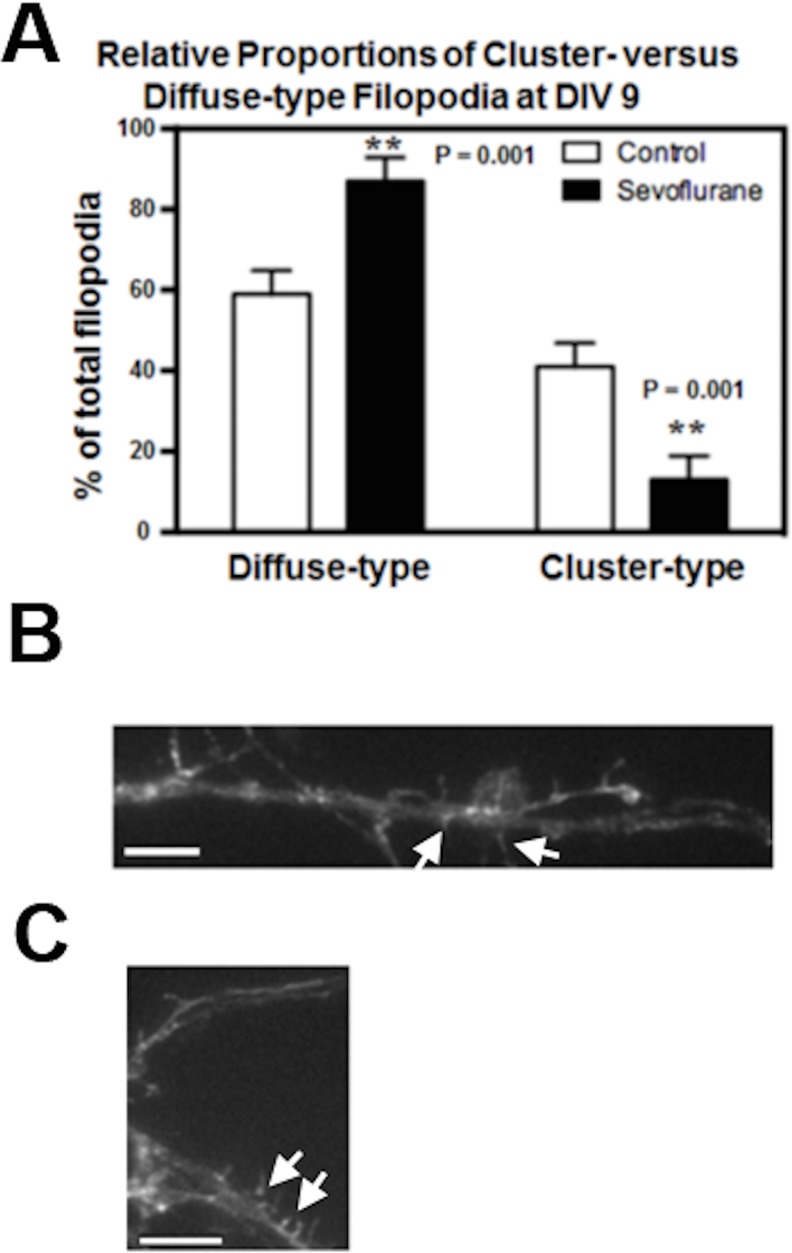
Sevoflurane alters the relative proportions of diffuse-type versus cluster-type filopodia in DIV9 developing mouse hippocampal neurons. (A) 3% sevoflurane for four hours on DIV7 caused an increase in diffuse-type and a decrease in cluster-type filopodia two days later compared to control unexposed hippocampal neurons. (B) representative fluorescent image of F-actin containing diffuse-type filopodia (arrows) in sevoflurane-exposed neurons. (C) representative fluorescent image of F-actin containing cluster-type filopodia (arrows) in control neurons. Phalloidin staining of F-actin was performed as described in Methods.(**: the difference between sevoflurane-exposed and unexposed neurons). N = 233 protrusions, similar results were obtained in two experiments. Calibration bars (B,C) are 5 microns in length.

**Fig 4:**
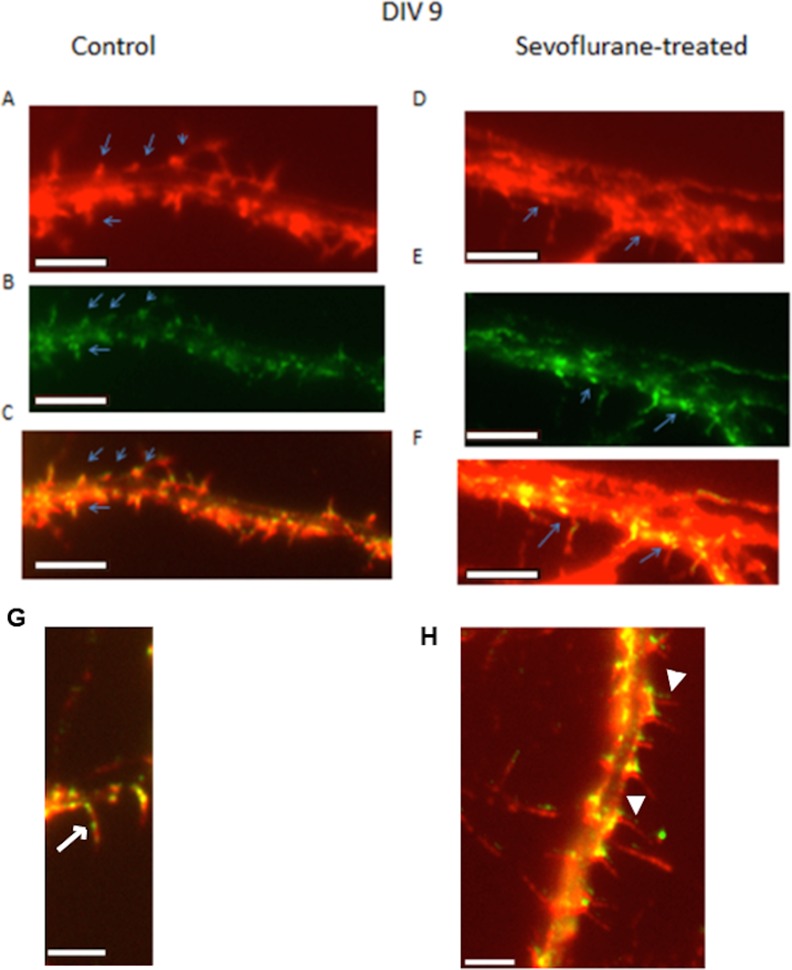
Dual F-actin (phalloidin) and drebrin immunoreactivity in control or sevoflurane-exposed DIV9 hippocampal neurons. (A-C) Control neurons stained with (A) phalloidin, (B) drebrin A/E polyclonal antibodies, or (C) in merged phalloidin/drebrin images demonstrate clustering of F-actin, and drebrin-IR in the protruding stalk or at the base of filopodial processes (arrows). (D-F) Sevoflurane-exposed neurons stained with (D) phalloidin, (E) drebrin antibodies, or (F) in merged phalloidin/drebrin images demonstrate clustering of F-actin, and drebrin IR predominantly in the submembranous regions of dendritic shafts (arrows) and much less co-clustering in filopodia stalks. Close-up views in merged image from control (G) neuron demonstrates the characteristic co-localization of drebrin-IR and drebrin-IR/phalloidin to the bases and mid-stalk filopodial locations (arrows, G); a merged image from sevoflurane-exposed (H) neuron reveals the absence of drebrin-IR in filopodial stalks, and thread-like, drebrin-IR projections emerging alongside drebrin-IR negative filopodia (arrowheads, H). Calibration bars are 5 microns (A-F) and 2.5 microns (G,H) in length.

Fluorescent images were acquired using a 100x NA 1.40 oil immersion objective and epifluorescene microscope, (Keyence BZ-9000; Osaka, Japan). Dendrite segments located distal to the first branch point from the cell soma and including the most distally-located, dendritic segments (2–5 per neuron) were randomly selected for analysis. Phalloidin-stained fluorescence images appear either in red, (Fig 1A) or following transformation using Image-J software (NIH, Bethesda, MD) in black and white, e.g. (Figs 1A, 1B, 2 and 3). Drebrin A is an actin-binding protein which plays a key role in early dendritic spine morphogenesis [16]. Since co-clustering of F-actin and drebrin is an early marker of synapse formation in filopodia or on the dendritic shaft [16], we performed dual immunostaining for F-actin and drebrin, e.g. (Fig 4), to test whether sevoflurane impairs F-actin/drebrin co-clustering which is a precursor to early synapse formation [16].

### Cell Viability Assays

The reduction of the tetrazolium MTT (3-(4, 5-dimethylthiazolyl-2)-2,5-diphenyltetrazolium bromide) to formazan in metabolically-active cells can be used to assess cell viability or cell proliferation. In post-mitotic, primary neuron cultures, cell viability following anesthesia was assessed using the MTT reagent (Sigma, St. Louis, MO) by modification of a previously described method [17]. Briefly, dissociated primary hippocampal neurons were plated at 100,000/mL in 6-well plates in serum-free B27/neurobasal medium. Following exposure to control or sevoflurane conditions on DIV7, the plates were either immediately processed for MTT reduction or maintained for 2 additional days at 37 degrees C in a 5% CO_2_/95% air incubator. Next, 75 μL MTT solution (5 mg/ml in PBS, pH 7.4) was added to each well containing 2 mL serum-free B27/neurobasal medium. The plates were incubated at 37 degrees C for 4 hours in the dark. The MTT solution in medium was aspirated off and1 mL MTT solvent (4 mM HCl, 0.1% Nondet P-40 (NP40) in isopropanol) was added to each well to solubilize the formazan crystals. The plates were shaken for 15 minutes at room temperature. The absorbance was measured with a plate reader (Synergy 2, Bio Tek Instruments) at test wavelength of 570 nm and reference wavelength 670 nm.

### Statistics

**Fig 5:**
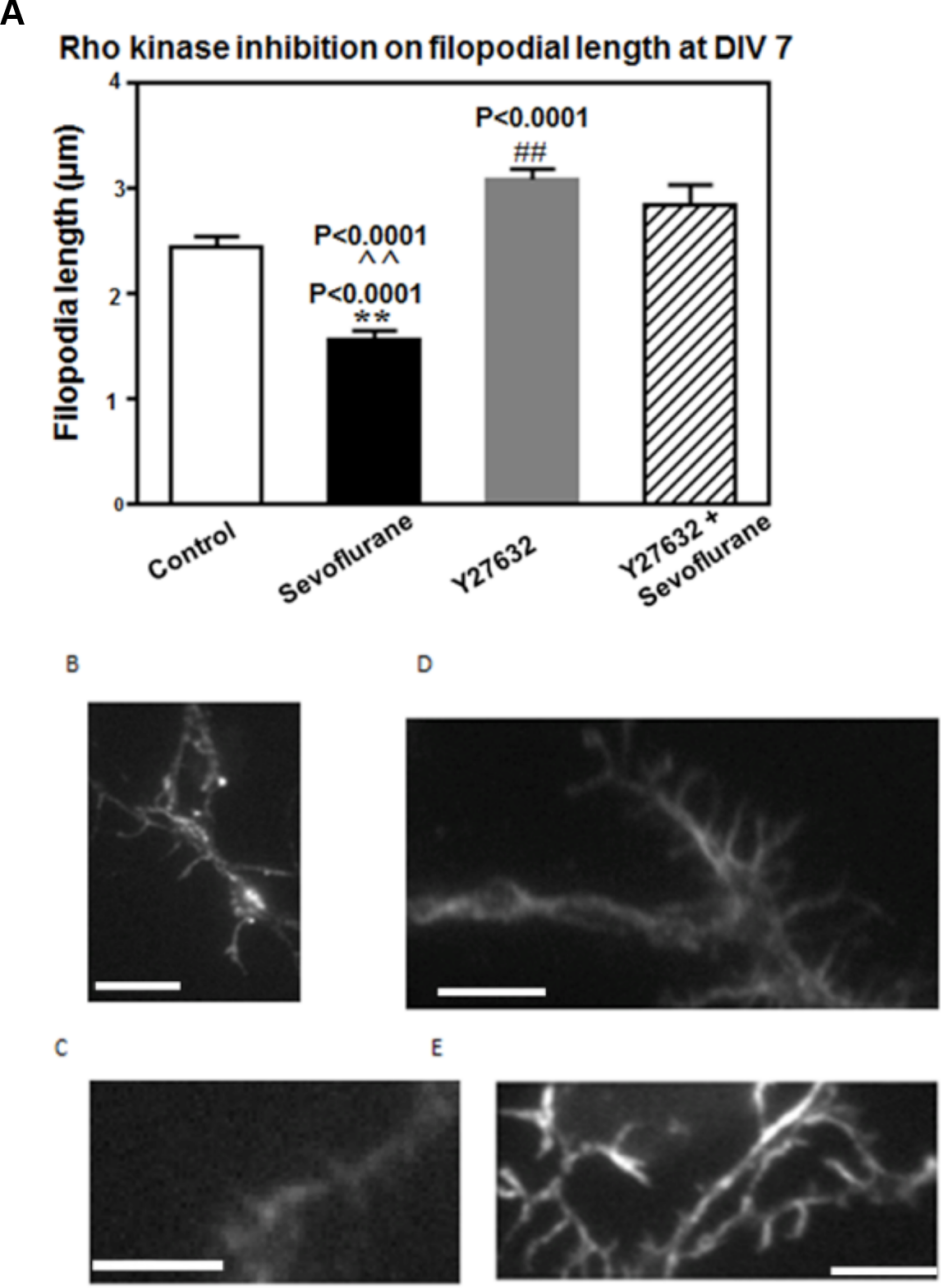
The Rho kinase inhibitor, Y27632, prevents sevoflurane-induced filopodial shortening in DIV7 immature hippocampal neurons. (A) 3% sevoflurane for four hours on DIV7 caused length-shortening in filopodia (solid bars) compared to control unexposed neurons (open bars). The length-shortening was completely prevented by co-incubating sevoflurane-exposed neurons with the selective Rho kinase inhibitor Y27632 (diagonal bars). Y 27632 alone (gray bars) increased filopodia length compared to control neurons. (**: the difference between sevoflurane-exposed and unexposed neurons; ^^ or the difference between sevoflurane-exposed in the presence or absence of Y27632; ## or the difference between control neurons in the presence or absence of Y27632. There was no significant interaction between Y27632 and sevoflurane. N = 318 protrusions, similar results were obtained in two experiments. Representative phalloidin images in (B) control, (C) sevoflurane-exposed, (D) Y27632-treated, or (E) Y2632 plus sevoflurane-exposed neurons. Calibration bars (B-E) are 5 microns in length.

**Fig 6:**
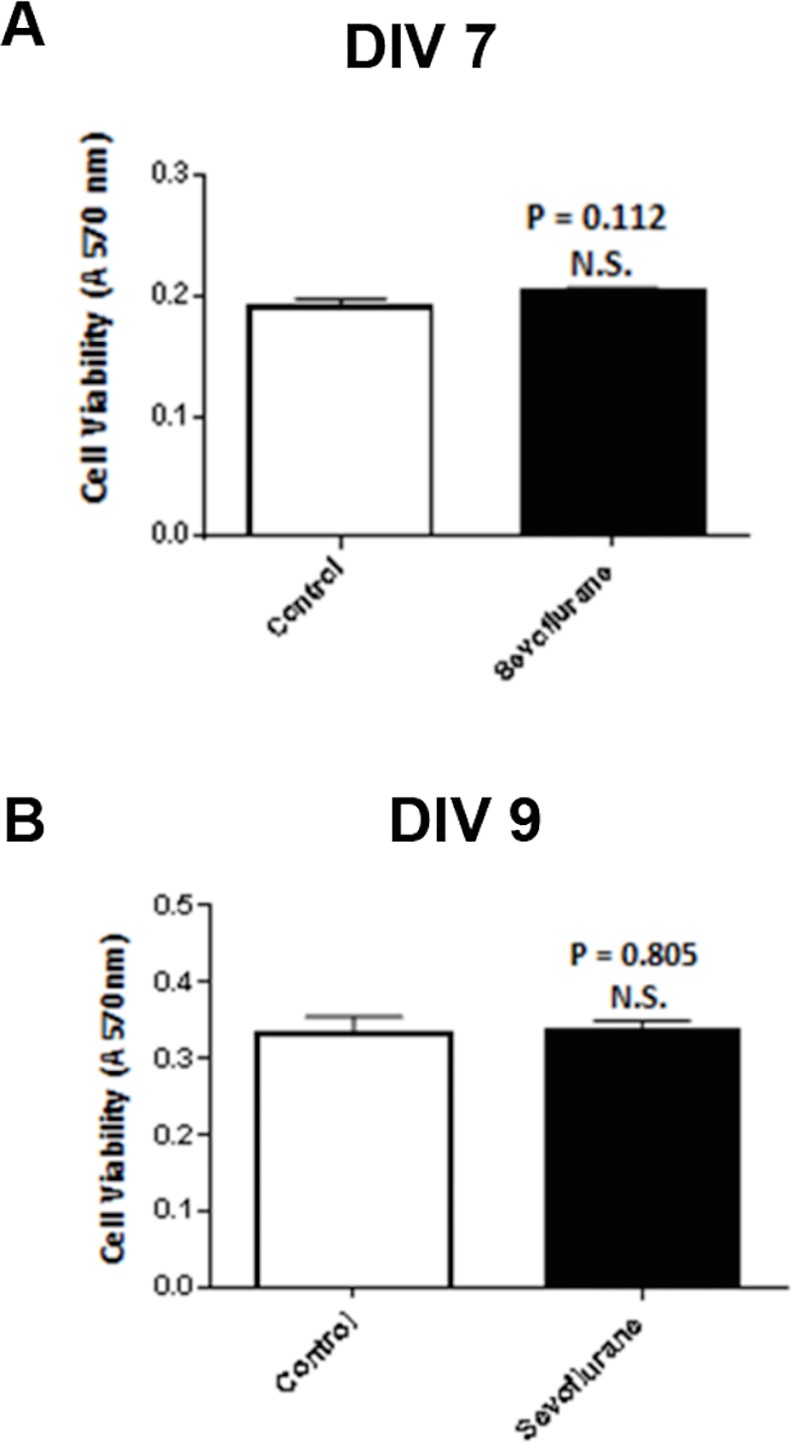
**Cell viability assessed by MTT assay in A) DIV 7 or B) DIV9 sevoflurane-exposed or control neurons**. 3% sevoflurane for four hours on DIV7 was not associated with a significant decrease in cell viability compared to control unexposed neurons examined at (A) DIV7 or (B) DIV 9. Each bar represents the mean + SE of 12 determinations in 6 wells. Similar results were obtained in two experiments.

ANOVA with repeated measurements was used to compare the means in control group, anesthesia, Y27632, and (anesthesia + Y27632) groups (Fig 5). Post-hoc analyses was used to compare the difference between anesthesia and control, anesthesia with or without Y27632, or Y27632 and control, cut-off alpha was Sidak-Bonferroni corrected (Fig 5). Student’s t-test was used to compare the difference between sevoflurane and the control group (Figs 1, 2, 3 and 6). Data were expressed as mean ± S.E. The significance testing was two-tailed, and P-values less than 0.05 were considered statistically significant. SAS software (Cary, NC) was used to analyze the data.

## Results

### Sevoflurane Causes Acute Length-Shortening in Immature Dendritic Protrusions

We first assessed the effects of sevoflurane on length of dendritic protrusions. A single 4-hour exposure to 3% sevoflurane caused acute significant filopodial length- shortening in DIV 7 hippocampal neurons: 1.58 ± 0.08 μ (sevoflurane, n = 47) versus 2.46 ± 0.09 μ (control, n = 105), (P < 0.001, Student-t test, Fig 1A). Thin spine length was also significantly reduced following sevoflurane exposure in DIV 7 neurons: 0.83 ± 0.08 μ (sevoflurane, n = 6) versus 1.37 ± 0.07 μ (control, n = 9), (P = 0.002, Student-t test, Fig 1B). Representative images of phalloidin-stained F-actin containing immature filopodia or thin spines in control (unexposed) or sevoflurane-exposed DIV 7 neurons are shown below the bar graphs in Fig 1A and 1B. In control neurons, long, thin, phalloidin-labeled protrusions (which lacked heads) were present along dendritic shafts at DIV 7 (Fig 1A, image to the left). In sevoflurane-treated neurons, phalloidin-labeled protrusions were much shorter, emerging only a short distance from dendritic shafts (Fig 1A, image to the right). Thin dendritic projections having a clearly-recognizable head (i.e. thin spines) were evident in DIV7 control neurons (Fig 1B, image to the left). In sevoflurane-treated, DIV7 neurons, the phalloidin-labeled thin spines were reduced in length owing to a markedly shortened neck (Fig 1B, image to the right). These results suggest that sevoflurane induces acute filopodial- or thin spine- shortening in DIV7 mouse hippocampal neurons by a mechanism which may involve F-actin depolymerization.

### Recovery of Dendritic Protrusion Length-Shortening following Sevoflurane

In neurons imaged two days after exposure to sevoflurane, mean filopodia length had fully recovered and was not significantly different compared to the mean levels in control, unexposed DIV9 neurons: 2.24 ± 0.26 μ, (sevoflurane, n = 38) versus 1.56 ± 0.18 μ, (control, n = 14) (P = 0.134, Student-t test, Fig 2A). Thin spine length in sevoflurane- exposed neurons significantly exceeded mean levels in control DIV 9 neurons: 1.67 ± 0.29 μ (sevoflurane, n = 9) versus 1.06 ± 0.05 μ (control, n = 15), (P = 0.014, Student-t test). These results suggest a compensatory increase in thin spine length two days after the treatment with sevoflurane. In control neurons, there was a non- significant decrease in mean filopodia length (1.95 *vs*. 1.56 microns; n = 51, P = 0.103) between DIV7 and DIV9 (not shown in Fig 2).

### Effect of Sevoflurane on Filopodia Maturation

During early development, immature dendritic protrusions on hippocampal neurons are predominantly filopodia having diffuse, F-actin staining [16]. Subsequently (from DIV 7–14) filopodia undergo a characteristic pattern of morphologic changes leading to the ontogeny of cluster-type filopodia, roughly 50% of which harbor synapses, i.e. synaptic filopodia [16]. Cluster-type filopodia are so named because they contain co-clusters of F-actin and the actin-binding protein drebrin A [16]. Drebrin A stabilizes F-actin and plays a key role in formation of the post-synaptic density (PSD) [16]. We therefore examined the relative numbers of diffuse- and cluster-type filopodia at DIV9: in unexposed (control) neurons, or neurons previously subjected to 4 hours of 3% sevoflurane on DIV 7. The number of diffuse-type filopodia in sevoflurane-exposed neurons significantly exceeded (~1.5-fold) those in control DIV 9 neurons (control: 59%, n = 70 *versus* sevoflurane: 87%, n = 99, P = 0.001, Student- t test) (Fig 3A). Cluster-type filopodia in control DIV9 neurons significantly exceeded (~ 3-fold) the numbers in sevoflurane-exposed neurons (control 41%, n = 49 *versus* sevoflurane: 13%, n = 15, P = 0.001, Student-t test) (Fig 3A). Representative images in sevoflurane-exposed DIV9 neurons demonstrate diffusely F-actin staining filopodia (arrows, Fig 3B). In contrast, control DIV 9 neurons show the characteristic clustering of F-actin, in the mid-stalk region and at the filopodial base which is typical of cluster-type filopodia at this stage in development (arrows, Fig 3B).

Dual drebrin/F-actin staining demonstrated that F-actin (phalloidin) (Fig 4A) and drebrin-IR (Fig 4B) clustered together (Fig 4C, merged image) at the bases and/or in the mid-stalk regions of filopodia in control DIV9 neurons. In contrast, in sevoflurane-exposed DIV9 neurons, F-actin (Fig 4D) and drebrin-IR clusters (Fig 4E) were localized to a region in the dendritic shaft not underlying a filopodium, and co-clusters (Fig 4F, merged image) localized predominantly in dendritic shafts as opposed to filopodial stalks. Close-up views of dendrites from control DIV9 neurons (Fig 4G, merged image) demonstrate the normal co-clustering of F-actin and drebrin-IR near the base(s) and in the midstalk filopodial region(s) characteristic of cluster-type filopodia [16] (arrows, Fig 4G). In sevoflurane-exposed DIV9 neurons, drebrin-IR clusters and F-actin/drebrin co-clusters (Fig 4H, merged image) did not appear in filopodial shafts, but were concentrated in submembranous dendritic shaft regions shifted away from the bases of filopodia (arrows, Fig 4H). Thread-like, drebrin-IR projections occurred alongside F-actin containing filopodia (arrowheads, Fig 4H) in sevoflurane-exposed, but not in control DIV9 neurons. The present results suggest that early sevoflurane exposure interferes with the formation of cluster-type filopodia in developing mouse hippocampal neurons which could adversely affect formation of synaptic filopodia, pending further investigation.

### Effect of Rho Kinase Inhibitor on Sevoflurane-Induced Filopodial Shortening

The small GTPase Rho A causes dendritic spine loss via activation of a signaling complex involving the Rho associated kinase, ROCK [18]. Rho A also inhibits activity in Rac, a distinct member of the small GTPase family of signaling molecules which promotes dendritic filopodial lengthening [19]. We next tested whether filopodia- and thin spine- shortening induced by sevoflurane requires RhoA/ROCK signaling pathway activation. Sevoflurane-exposed or unexposed DIV 7 neurons were incubated in the presence or absence of (10 μM) concentrations of the selective ROCK inhibitor Y27632. Mean filopodial length differed significantly (one-way ANOVA, F = 28.57, P < 0.0001) among the four groups of DIV 7 neurons [control with or without Y27632, and sevoflurane-exposed with or without Y27632] (Fig 5A). Sevoflurane neurons had significantly shorter filopodia (1.58 μm, n = 47 *versus* 2.46 μm, n = 105, P < 0.0001) compared to control unexposed neurons (Fig 5A). Y27632 in the presence of sevoflurane (2.85 μm, n = 29) significantly (P< 0.0001) prevented filopodia shortening compared to sevoflurane alone. Y27632 alone (3.09 μm, n = 137) caused significant (P < 0.0001) filopodial lengthening compared to the control neurons (Fig 5A). There was no significant interaction effect of sevoflurane and Y27632. Representative images of DIV7 filopodia in control, sevoflurane-exposed, Y27632 alone, or sevoflurane in the presence of Y27632 are shown in Fig 5B, 5C, 5D and 5E, respectively.

### Effect of Sevoflurane on Neuron Viability

A single four hour exposure to 3% sevoflurane on DIV7 had no significant effect on hippocampal neuron viability assessed in MTT assays performed on DIV 7 (Fig 6A) and on DIV 9 (Fig 6B).

## Discussion

The current findings are the first to suggest that early exposure in mouse hippocampal neurons to clinically-relevant concentrations of sevoflurane substantially impaired filopodial and thin spine length and altered the normal maturational sequence in a subset of filopodia. The precise mechanism of sevoflurane’s actions on immature dendritic protrusions is not yet clear. The complete rescue of filopodial or spine length-shortening by the selective Rho kinase inhibitor Y27632 demonstrated here suggests involvement (in part) of the RhoA/ROCK signaling pathway in the mechanism of sevoflurane neurotoxicity. These findings are consistent (in part) with a prior report that brief isoflurane exposure (2 vol% for 60 minutes) caused reversible spine shrinkage in mature (day in vitro 21) cultured rat hippocampal neurons through a mechanism involving F-actin depolymerization [19]. In contrast, Lemkuil et. al. [13] reported that 1.4% isoflurane for 4 hours in postnatal day 4–7 mouse hippocampal neurons caused neuroapoptosis via a mechanism involving RhoA activation, F-actin depolymerization and cleaved caspase-3 activation. In the present study, neuron viability (assessed morphologically and biochemically) was unaffected by 3% sevoflurane for four hours in DIV7, immature mouse hippocampal neurons. Taken together with a report by Wang et. al. [10] that sevoflurane reduced synaptogenesis (in neonatal rodent neurons) and resulted in long-lasting behavioral abnormalities without causing neuroapoptosis, the findings point to a selective synaptic mechanism of sevoflurane neurotoxicity. Dysregulation of Rho GTPase(s) plays a crucial role in impairment of early synapse formation in developing neurons in human diseases characterized by severe behavioral abnormalities. For example, in mice harboring an inactivating mutation in the human lissencephaly disease-causative Lis gene, cultured DIV7 hippocampal neurons exhibited decreased synaptogenesis associated with filopodia length-shortening, and the latter abnormality was rescued by treatment with Y27632 [20].

The Rho family of small GTPases is critical in regulating the actin remodeling in dendritic spines and immature filopodia which underlies synapse formation or synapse loss [21]. Specific defects in molecular regulation of the RhoA/Rho kinase signaling pathway, e.g. inactivating mutation in a Rho GTPase- activating protein, cause specific X-linked form(s) of human mental retardation [22] characterized by abnormal dendritic spine morphology and/or density [23]. Through its effects on actin cytoskeletal remodeling in spines, RhoA/Rho kinase activation was previously associated with dendritic spine shrinkage and synapse loss [20, 21, 24]. Sevoflurane is the most widely- used volatile general anesthetic in the pediatric population. Thus the current findings are relevant to the safety of sevoflurane (and possible mechanisms of its anesthesia neurotoxicity) in infants and young children.

Volatile anesthetic drugs including sevoflurane suppress glutamatergic excitatory neurotransmission through both pre-synaptic [25] and N-methyl D-aspartate receptor- mediated (NMDAR) post-synaptic mechanisms [25]. Recovery from sevoflurane anesthesia (in the current study) was associated with a significant increase in the mean length in thin dendritic spines in DIV 9 neurons. This might be consistent with a compensatory increase (following sevoflurane) in excitatory NMDA receptor-mediated glutamatergic synaptic input which promotes dynamic filopodia formation in immature developing hippocampal neurons [26]. NMDAR- mediated excitatory activity can alter the distribution of key cytoskeletal components in dendritic spines. For example, in spines undergoing learning, drebrin redistributed to dendritic shafts following NMDA- mediated excitatory activity [27]. Thus it is possible that activity-dependent or -independent changes in cytoskeletal proteins may have played a role in the absence of drebrin IR in a subset of filopodia stalks in DIV9 sevoflurane-exposed neurons (Fig 4F and 4H) which yet contained abundant drebrin-IR in dendritic shaft regions.

Drebrin plays key roles in spine morphogenesis [16], and in activity-dependent, spine shape changes underlying learning [28]. Drebrin has binding sites for F-actin, and it also binds the microtubule-binding protein EB3 located at the plus end of dynamic microtubules [29]. Prior reports indicate that microtubule (MT) invasion of filopodia and spines is associated with increased postsynaptic density-95 in developing mouse hippocampal neurons [30]. MT invasion of spines was enhanced by local spine Ca ^2+^ release, or synaptic NMDAR activity and it was suppressed by decreased spine F-actin content or reduced drebrin A expression [30]. Thus sevoflurane anesthesia may have caused a loss of microtubule dynamics in early dendritic spines (in part) as a result of decreased F-actin filopodia content or preferential drebrin A localization to dendritic shafts as opposed to spines or filopodia. One possible explanation for the persistent absence of drebrin-IR in a subset of DIV9 neurons, two days after sevoflurane exposure, is that large, intensely-staining F-actin/drebrin co-clusters localized at filopodia bases may have posed a physical barrier to microtubule invasion of filopodia. In prior studies, RhoA/Rho kinase activation in growth cone filopodia caused myosin I-mediated actomyosin contraction at the base of filopodia resulting in consolidation of F-actin together with dynamic microtubules [31]. Microtubule invasion of spines was shown to be critically-dependent on polymerization occurring very close to the base of filopodia [30] suggesting it is particularly sensitive to biochemical processes occurring in the filopodia stalk [30]. In contrast, non-invading dynamic MT were reported to extend substantial distances from dendritic shafts [30] perhaps accounting for the thread-like drebrin IR- positive, F-actin negative projections which emerged alongside drebrin IR -negative filopodia (green, arrowheads, Fig 4H).

Drebrin-IR clusters occurring in dendritic shaft regions (Fig 4H) were laterally-displaced from peak F-actin clusters located near the bases of filopodia in sevoflurane-exposed neurons. Another possible mechanism may have involved sevoflurane- induced limited disassembly in dendritic shaft microtubules. The Rho A/Rho kinase signaling pathway was reported to cause phosphorylation of the dendritic microtubule associated protein, MAP2 [32]; MAP2 phosphorylation destabilizes microtubules [32]. Thus drebrin binding to microtubule -binding EB3 at the plus-end of partially disassembled dendritic shaft microtubules might interfere with drebrin’s ability to bind F-actin filaments that project into the filopodial stalk.

Although the precise mechanisms are unknown, filopodia which lacked drebrin and invading MTs are unlikely to form postsynaptic densities required for synapse formation. A decrease in synaptic filopodia numbers might adversely affect network electrical activity at a critical stage when spontaneous synchronized electrical activity normally occurs [33] and is thought to be important in shaping synaptic connectivity in the maturing network [34–36].

Prior studies indicate that drebrin loss from spines preceded synapse loss in humans with Alzheimer’s disease or Down’s syndrome [37] and it correlated with memory loss in mouse models of Alzheimer’s disease [38]. Taken together, these findings are consistent with the possibility that absence of drebrin-IR localization in a subset of maturing filopodia might have a causative role in decreased synapse formation occurring during a critical developmental period when the functioning hippocampal neuron network is becoming hard-wired.

Our study has several limitations. First, it was based (in part) on post-hoc analyses in subgroups of neurons in which case the results need to be interpreted cautiously. Second, our *in vitro* model system did not allow for an extrapolation of the observed sevoflurane-induced alteration(s) in dendritic spine morphology to possible effects on behavior. Zhou et. al. [39] reported that a single exposure (2.3% sevoflurane for 5 hours) in 6-day-old Cynomolgus monkeys did not alter protein expression levels of post synaptic density (PSD)- 95, or drebrin; nor significantly affect the monkeys’ performance in specific learning and memory tasks, when assessed at 7 months of age. Yet in neonatal mice, multiple exposures to sevoflurane caused learning and memory impairment which was lessened by environmental enrichment [40]. One possibility (consistent with the present findings) is that experience-dependent activity promotes synaptic strengthening which can help overcome a specific kind(s) of synaptic deficit induced by sevoflurane. There may be unknown species differences in how immature mouse or non-human primate neurons respond to sevoflurane anesthesia which are important in determining the safety of sevoflurane administration during early human childhood and infancy.

The Rho kinase inhibitor Y27632 not only completely rescued filopodial- and thin spine-shortening induced by sevoflurane, but it also significantly increased filopodia length compared to control neurons. More study is needed to determine whether Y27632 may restore drebrin clustering within filopodial stalks in sevoflurane-exposed neurons or prevent other structural abnormalities in early filopodia which might negatively affect synaptogenesis.

## Conclusions

In conclusion, we have demonstrated that anesthetic sevoflurane can reduce the length of filopodia and thin spines in DIV7 neurons, decrease the number of cluster-type filopodia in DIV9 neurons, and impair the normal localization of drebrin immunoreactivity to filopodia stalks in a subset of DIV9 neurons. Rho kinase inhibitor Y27632 rescued the sevoflurane-induced reduction in the length of filopodia and thin spines. These novel data suggest that sevoflurane may affect the formation of synapses via one or more Rho kinase-associated mechanisms. These findings have provided a model system for better understanding of the effects of anesthetics on synapse formation and function, which would promote more research to investigate the mechanisms of anesthesia-neurotoxicity in infants and children.

## Supporting Information

S1 Data Raw data Fig 1A. (XLSX)

S2 Data Raw data Fig 1B. (XLSX)

S3 Data Raw data Fig 2A. (XLSX)

S4 Data Raw data Fig 2B. (XLSX)

S5 Data Raw data Fig 3A. (XLSX)

S6 Data Raw data Fig 5A. (XLSX)

S7 Data Raw data Fig 6A. (XLSX)

S8 Data Raw data Fig 6B. (XLSX)
